# Predictive ability of novel glycovariant biomarkers of CA125 and CA15-3 up to three years prior to ovarian cancer diagnosis: a population-based case-control study

**DOI:** 10.1186/s13048-026-02143-5

**Published:** 2026-05-29

**Authors:** Hanna Roos Alexander, Shamima Afrin Ruma, Shruti Jain, Eva Lundin, Per Liv, Pernilla Israelsson, Kim Pettersson, Annika Idahl

**Affiliations:** 1 Department of Clinical Sciences, Obstetrics and Gynecology, Umeå University, Universitetstorget 4, Umeå, 901 87 Sweden; 2 Department of Life Technologies and FICAN West Cancer Centre, University of Turku, Turku, Finland; 3 Department of Medical Biosciences, Pathology, Umeå University, Umeå, Sweden; 4 Department of Public Health and Clinical Medicine, Umeå University, Umeå, Sweden; 5 Department of Diagnostics and Intervention, Oncology, Umeå University, Umeå, Sweden

**Keywords:** Epithelial ovarian cancer, Biomarkers, Glycovariant, CA125, Early detection, Prospective samples

## Abstract

**Background:**

Nanoparticle immunoassays for CA125 and CA15-3 glycovariants are promising tools for epithelial ovarian cancer (EOC) and borderline ovarian tumor (BOT) early detection.

**Method:**

This retrospective population-based study utilized prospective (< 3 years) plasma samples from 110 cases and 440 controls. Sialyl-Thomsen-nouveau (STn) antibody and macrophage galactose-type lectin (MGL) to detect cancer antigen 125 (CA125) and 15 − 3 (CA15-3) glycoforms were evaluated, using CA125, CA15-3, and HE4 enzyme immunoassay (EIA) references. The area under the receiver operating characteristic curve (AUC), partial AUC (pAUC) at specificities 0.9–1.0, and sensitivity (SN) at 0.98 specificity (SP) for detecting EOC/BOT were calculated.

**Results:**

The single marker with the highest point estimate for pAUC and SN at 98% SP was CA125^EIA^ (pAUC 0.71 (0.66, 0.76), SN at 98% SP 0.29 (0.17–0.42 combinations)). The CA15-3^STn^ + CA125^EIA^ and CA125^EIA^ + HE4^EIA^ improved the detection rate point estimates and provided the highest pAUC values (CA125^EIA^ + CA15-3^STn^: 0.75 (0.70–0.80); CA125^EIA^ + HE4^EIA^: 0.71 (0.66–0.77)). For 0–<1, 1–<2 and 2–<3 years lag time, CA125^EIA^ and the CA125 glycovariants provided similar pAUC and SN at 98% SP, with largely overlapping confidence intervals.

**Conclusion:**

This case-control study, which used plasma samples collected up to three years before diagnosis of EOC or BOT, did not provide evidence of a higher discriminatory capacity of the glycovariant biomarkers compared with the clinically established biomarker CA125^EIA^ in asymptomatic women. Increased plasma volumes and larger cohorts are suggested in future studies.

**Supplementary Information:**

The online version contains supplementary material available at 10.1186/s13048-026-02143-5.

## Introduction

Epithelial ovarian cancer (EOC) is the most lethal gynecological malignancy [1]. While the five-year survival rate of patients with early-stage EOC (International Federation of Gynecology and Obstetrics (FIGO) I and II) is about 92%, it is only 29% in patients with advanced-stage EOC (FIGO III and IV) [2]. Borderline ovarian tumors (BOT) represent an intermediate category between benign cystadenomas and EOC, making them a distinct histological and clinical entity. They account for approximately 10–20% of all epithelial ovarian tumors and are characterized by atypical epithelial cell proliferation without stromal invasion [3]. BOT are also considered precursors of some histological subtypes of EOC [4]. 

Roughly 60% of ovarian cancers are diagnosed in an advanced stage. Ovarian cancer generally does not exhibit specific early symptoms, and diagnostic procedures have suboptimal discriminatory capacity; together, these result in delayed cancer diagnoses as well as morbidity due to overtreatment. Earlier detection has been achieved using multimodal screening in women with average population risk, although no randomized trial has shown a mortality benefit [5]. Therefore, ovarian cancer screening is not currently recommended in women at average population risk [6]. It is important to identify reliable biomarkers for ovarian cancer; whether individually or in combination with biomarkers already in use, to improve sensitivity and specificity for detecting EOC and BOT at an early stage or in a screening context.

In 1981, Bast et al. described the high molecular mucinous glycoprotein CA125, also known as MUC16, for the first time [7]. CA125 is found on the surface of EOC cells and is the most widely used marker for diagnosing EOC through quantitative detection in the serum of patients with EOC. CA125 and human epididymis protein 4 (HE4) are the two biomarkers that are in clinical use for diagnosing EOC today. However, findings from randomized screening trials and prospective population cohorts have shown insufficient sensitivity and specificity on the part of both CA125 and HE4 [5, 8, 9]. Another biomarker found to be elevated in ovarian cancer is cancer antigen 15 − 3 (CA15-3), also known as MUC1, although it is not superior to CA125 or HE4. CA15-3 is primarily established as a tumor marker for breast cancer and is not in clinical use for EOC detection [10, 11].

Substantial effort is being expended in the search for additional protein biomarkers that, individually or in combination with CA125 and other markers, could enhance the sensitivity and specificity of tests for detecting ovarian cancer at an earlier, curable stage. So far, none has reached the market.

The majority of the tumor markers in use today are glycosylated proteins [12]; in many types of human cancer, these undergo aberrant glycosylation during the malignant process [13].

Detecting specific glycosylation patterns on a protein marker—rather than measuring protein levels, as is currently done—could increase the specificity and sensitivity of biomarker assays [14]. Lectins are a highly diverse group of proteins that bind to carbohydrate structures [15] and offer the possibility of detecting tumor-specific glycan structures. Compared to conventionally used antibodies, lectins generally have low affinities, which has limited their use in diagnostic applications.

The use of fluorescent nanoparticles, on which lectins and low-affinity antibodies are immobilized, substantially increases binding strength and the potential of ultra-sensitive bioaffinity applications [16]. For the detection of cancer-associated glycoprotein glycoforms, tumor marker targets are captured from the sample using specific lectins or antibodies: recombinant human macrophage galactose-type lectin (MGL), and Sialyl-Thomsen-nouveau (STn) antibody [17]. This new method has been shown to have improved sensitivity at high specificity across several histotypes in blood samples of women with ovarian carcinoma and BOT compared to the conventional CA125 enzyme immunoassay (CA125^EIA^), which is currently in routine clinical use [18]. Glycovariants of CA15-3 have also been reported to be elevated in ovarian cyst fluid [19]. However, while this new method of lectin-assisted detection of tumor-specific glycan structures on glycoprotein biomarkers has shown promising results regarding early diagnosis of ovarian carcinomas, its diagnostic performance prior to diagnosis of EOC and BOT is not known.

Whereas our previous studies have shown the superiority of CA125 and CA15-3 glycovariants for detecting EOC over benign ovarian conditions, this study aimed to compare the diagnostic accuracy of CA125 and CA15-3 glycoforms, aided by STn- and MGL-coated nanoparticles, with conventional CA125^EIA^ using specimens prospectively collected up to three years prior to diagnosis of EOC and BOT and (for comparison) apparently healthy controls.

## Methods

### Study design

This was a retrospective, population-based case-control study nested within the Northern Sweden Health and Disease Study (NSHDS) that used prospectively collected plasma samples.

#### Ethics

This study was approved by the Regional Ethical Review Authority in Umeå, Dnr. 2017-376-31.

### Study population

#### Case-control selection

**Fig. 1:**
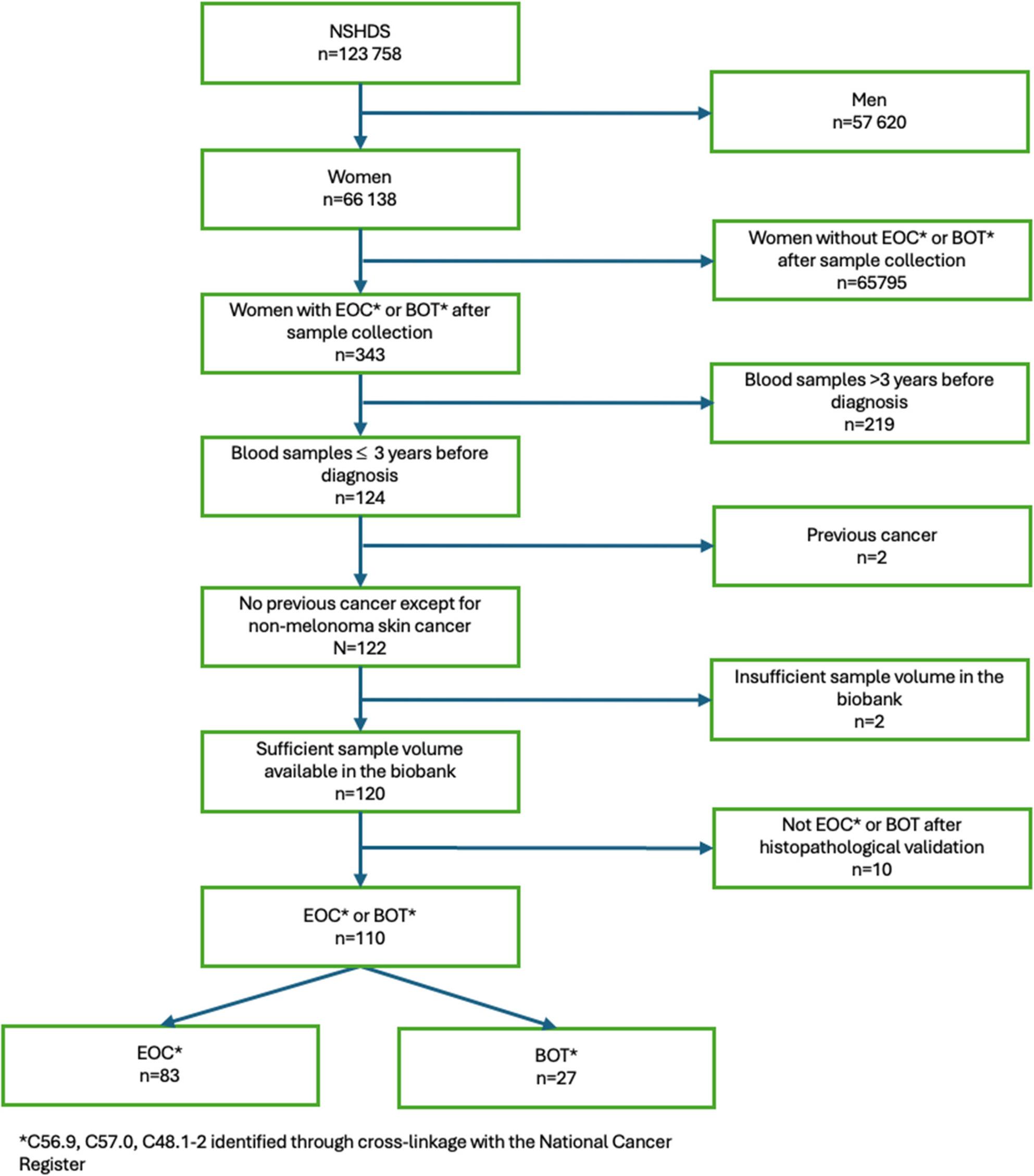
Flowchart illustrating the case-selection of the study population Flowchart illustrating the case-selection of the study population derived from the Northern Sweden Health and Disease Study (NSHDS) 1990–2017. Women diagnosed with EOC or BOT were identified through the National Cancer Register. Eligible participants were required to have donated a prediagnostic blood sample within three years prior to diagnosis and to have no history of previous cancer except non-melanoma skin cancer. After applying all inclusion and exclusion criteria, the final cohort consisted of women with validated diagnoses and available pre-diagnostic plasma samples. Abbreviations: BOT: Borderline Ovarian Tumor, EOC= Epithelial Ovarian Cancer, NSHDS= Northern Sweden Health and Disease Study

In total, 110 cases were selected. All ovarian cancer and borderline ovarian tumor cases (primary site ovary C56.9, fallopian tube C57.0, peritoneum C48.1-2), identified through cross-linkage with the National Cancer Register, who also had prospective plasma samples within 3 years prior to diagnosis in the NSHDS- biobank, were included in the study (Fig. 1). For each of the 110 cases, four controls (*n* = 440) were randomly selected among appropriate risk sets and matched on age (+/- 6 months) and time of blood draw (+/- 3 months). The cases and controls had no previous cancer, except non-melanoma skin cancer, at the time of diagnosis of the index case, and the controls were alive at the time of diagnosis of the index case.

If there were several prospective plasma samples within three years prior to diagnosis, the sample closest to diagnosis was selected.

#### Sample collection

The blood samples were collected at health care facilities as part of a non-selective health-intervention program offered to all inhabitants in the Västerbotten county of Sweden, between 1990 and 2017. Prior to diagnosis and following the obtaining of informed consent, patients who were subsequently diagnosed with ovarian cancer prospectively donated plasma samples. These were collected as part of either the Northern Sweden Monitoring Trends and Determinants in Cardiovascular Disease (MONICA) study (8.3%), the Mammography screening cohort (40.5%) or the Västerbotten Intervention Programme (VIP) (51.2%) within the NSHDS at Umeå University [20]. The blood samples were drawn after 5 min of rest and more than 8 h of fasting for most (83%) subjects. The blood samples used in this study were collected in EDTA tubes, and aliquots of plasma, buffy coat, and erythrocytes were processed and frozen (− 20 °C) within 1 h of collection. Within a week, the tubes were transported to − 80 °C freezers at a central storage facility [20]. In this study, EDTA plasma was used.

#### Classification of clinical stage and morphology

Histopathological diagnoses were validated by histopathological report review by experienced senior consultant gynecologic pathologists, and adapted to the 2014 World Health Organization (WHO) pathologic classification of gynecologic tumors [21]. Cases that had been classified before 2014 as a serous grade 2 carcinoma were reclassified as either high-grade (HGSC) or low-grade (LGSC) after reassessment of tumor slides to avoid/reduce the risk of misclassification. Tissue samples with metastases to the ovary with primary origin of cancer or borderline tumor elsewhere were excluded from the analysis. Women with BOT or carcinomas with the primary site in the ovary C56.9, fallopian tube C57.0, and peritoneum C48.1-2 (according to ICD-O/2) were included. The stage classifications according to FIGO 2014 were retrieved by manual review of medical records.

### Laboratory assays

#### Materials

OVCAR-3 cell line purified CA125, anti-CA125 (Ov185), and anti-CA15-3 (Ma552) monoclonal antibody (mAb) that specifically recognize different protein epitopes were made available by Fujirebio Diagnostics (Gothenburg, Sweden), as was STn1242 mAb that recognizes sialylated Tn antigen. Yellow streptavidin (SA)-coated low-fluorescence microtitration plates, wash buffer, and assay buffer were obtained from Uniogen Oy (Turku, Finland). The recombinant human lectin MGL-Fc (also known as CLEC10A) was purchased from Sino Biologicals Inc. (Beijing, China). Europium (III)-chelate doped Fluoro-Max™ polystyrene nanoparticles (95 nm in diameter, 30000 chelates per particle, Eu^+ 3^-NP) were purchased from Seradyn Inc. (Indianapolis, IN).

#### Glycovariant assays

Three biomarkers were tested: CA125^STn^, CA15-3^STn^, and CA125^MGL^. Previous studies [12–14] have provided details on the glycovariant assays for CA125 and CA15-3. To summarize, streptavidin-coated low-fluorescence microtiter wells (Kaivogen Oy) were used, and biotinylated capture antibodies (Ov185 Fab2 for CA125, and Ma552 Fab2 for CA15-3) were immobilized onto the wells at a concentration of 50 ng/25 µl/well in assay buffer (including 9 mM CaCl_2_). This immobilization step occurred at room temperature (RT) for 60 min without shaking. After two washes, 25 µl of either a standard or diluted plasma sample (1:10 for CA125 and 1:40 for CA15-3) in assay buffer (including 300 mM NaCl, 100 mM CaCl_2_, and 5 µg/ml MAK33 poly blocker) was added in triplicate and incubated for 60 min at RT with shaking. For the tracer, 25 µl of assay buffer (same as the capture buffer) containing 1 × 10^7^ Eu^+ 3^-NPs coated with MGL or anti-STn mAb was added to each well and incubated for 1 h at RT with shaking. Following the incubation, the wells were washed six times with wash buffer. The time-resolved fluorescence (TRF) of Eu^+ 3^ was measured (λ_ex_: 340 nm; λ_em_: 615 nm) from dry wells using the Hidex Sense Multi-Technology Microplate Reader (Hidex Oy, Turku, Finland).

To create standards for the CA125 glycovariants (both MGL and STn), OVCAR3 cell line purified CA125 stock solution, provided by Fujirebio, was used to prepare standards, ranging from 2 to 500 U/ml. The limit of detection (LOD) was 1.6 U/ml for CA125^STn^ and 1.2U/ml for CA125^MGL^. For the CA15-3^STn^ standards, ascites from a patient with EOC containing 300 U/ml of CA15-3 were used; the LOD was 1.3 U/ml. Standards and samples were added in triplicate, and the coefficient of variance (CV%) was calculated. Additionally, each plate included two separate controls of lower and higher concentrations to facilitate observation of day-to-day control variation.

#### Reference assay

Conventional EIAs of CA125, CA15-3, and HE4 were run as reference. Fujirebio CanAg CA125^EIA^, CanAg CA15-3^EIA^, and CanAg HE4^EIA^ kits were used according to the manufacturer’s instructions for conventional assays. CA125^EIA^ and HE4 were selected as reference biomarkers because they are widely accepted and used in clinical practice for ovarian cancer diagnosis.

While CA15-3 is primarily used for breast cancer diagnosis, it has also been reported as elevated in ovarian cancer.

### Statistical analysis

For each of the markers, as well as for selected combinations of markers, the area under the receiver operating characteristic curve (AUC), the partial AUC (pAUC) at specificity levels of 0.9–1.0, and the sensitivity at 0.98 specificity (sens_spec0.98_) for diagnosing ovarian cancer were calculated. AUC, pAUC, and sens_spec0.98_ values for the markers were evaluated using logistic regression models, where the markers were transformed using the fifth root to reduce skewness and entered into the model using restricted cubic splines with three knots, placed at the 10th, 50th, and 90th percentiles. The splines were used to account for non-linearity in associations. Adjustments were also made for two matching variables—age at diagnosis and time from sample to diagnosis—to account for the case-control structure of the data [22].

The estimates of AUC, pAUC, and sens_spec0.98_ were internally validated by correcting for overfitting using bootstrapping with 200 replicates. The bootstrapping was performed following the procedure suggested by Harrell et al. [23], but was based on selection with replacement of cases along with their corresponding controls in order to preserve the case-control structure of the data. Confidence intervals were estimated using a nested bootstrapping procedure (i.e. an inner bootstrap of the previously described validation was performed within each of 500 outer bootstrap replicates). Percentile confidence intervals were determined from the 500-bootstrap simulated and validated values of AUC, pAUC, and SN at 98% SP based on 500 outer bootstrap replicates. AUC, pAUC, and SN at 98% SP were also calculated for the subgroups of invasive cancer, BOT, HGSC, and for women ≥ 51 years of age. Due to the small sample size, the subgroup results were not bootstrap validated.

Confidence intervals for unvalidated AUC, pAUC and sens_spec0.98_ were estimated from 1000 bootstrap replicates.

Locally estimated scatterplot smoothing (Loess) regression curves were used to visualize the average levels of the different markers, separately for cases and controls, as a function of the time between blood-sample collection and diagnosis date of the cases. The stability of ranking of the markers based on highest pAUC was examined using a bootstrap procedure for 200 simulated bootstrap samples obtained from sampling cases, including the corresponding matched controls, with replacement. For each bootstrap sample, pAUC values for the single markers and marker combinations were estimated and ranked from highest to lowest. Thus, the procedure resulted in a sample distribution of 200 estimated ranking positions for each marker/marker combination. The spread of the rankings of each marker/marker combination, as well as the median and quartiles of the rankings, were then visualized.

## Results

### Study population

In total, 120 cases wherein plasma samples were collected as part of the NSHDS less than three years prior to EOC or BOT diagnosis were identified. Of these, ten cases were excluded: two due to missing pathological reports, three were benign cases, two were cases of malignancies other than ovarian cancer, and three were cases of non-epithelial ovarian cancer (two granulosa cell tumors and one germ cell tumor). Following this process, 110 cases thus remained (Fig. 1).

The final study population consisted of 110 cases wherein plasma samples were collected up to three years before ovarian cancer diagnosis. The median age of the cases was 60.4 (30.6–74.8) years. Forty cases (36.4%) were collected 0–<1 year before diagnosis, 40 (36.4%) cases were collected 1–<2 years before diagnosis, and 30 (27.3%) cases were collected 2–<3 years before diagnosis. Epithelial ovarian cancer was diagnosed in 83 (75.5%) cases, and BOTs in 27 (24.5%) cases. The most common histotype was HGSC (*n* = 39, 47%), followed by endometrioid carcinoma, mucinous carcinoma, clear cell carcinoma, and undifferentiated carcinosarcoma. The majority were diagnosed as late stage FIGO III and IV (*n* = 46, 55.4%). Further details of the study population are provided in Table 1.

**Table 1:** Characteristics of the study population

	Case	Control
	*n* = 110	*n* = 440
Age at diagnosis		
Median (range)	60.4 (30.6–74.8)	60.2 (30.4–75.0)
<50 years, n (%)	7 (6.4)	29 (6,6)
≥50 years, n (%)	103 (93.6)	411 (93.4)
Years prior to diagnosis n (%)		
0 – <1	40 (36.4)	160 (36.4)
1 – <2	40 (36.4)	160 (36.4)
2 – <3	30 (27.3)	120 (27.3)
BMI		
Median (range)	26.2 (17.8–58.9)	25.9 (16.9–43.3)
Missing, n (%)	9 (8.2)	27 (6.1)
Smoking		
Current	29 (26.4)	100 (22.7)
Non-smoker/former smoker	79 (71.8)	327 (74.3)
Missing	2 (1.8)	13 (3)
Invasive EOC	83 (75.5)	
BOT	27 (24.5)	
Histology EOC, n (%)		
HGSC	39 (47.0)	
LGSC	10 (12.0)	
Endometroid	13 (15.7)	
Clear cell	7 (8.4)	
Mucinous	11 (13.3)	
Carcinosarcoma	1 (1.2)	
Undifferentiated	2 (2.4)	
Histology BOT, n (% of BOT)		
Borderline serous	18 (66.7)	
Borderline mucinous	9 (33.3)	
Clinical stage^a^		
Early stage	36 (43.4)	
stage I	31 (37.3)	
stage II	5 (6.0)	
Late stage	46 (55.4)	
stage III	36 (43.4)	
stage IV	10 (12.0)	
Missing	1 (1.2)	

*Abbreviations*: *BMI* Body mass index, *BOT* Borderline ovarian tumor, *EOC* Epithelial ovarian cancer, *HGSC* High-grade serous cancer, *LGSC* Low-grade serous cancer, *n* Number

^a^Clinical stage according to FIGO classification

### Diagnostic performance of glycovariant assays prior to diagnosis

The performance of each biomarker for all tested cases over the entire period of three years prior to diagnosis is shown in Table 2.

**Table 2:** Nanoparticle-aided detection of CA125 and CA15-3 glycovariants, and EIA biomarkers in ovarian cancer Samples collected prospectively up to three years prior to epithelial ovarian cancer (*n* = 83) or borderline ovarian tumor (*n* = 27) diagnosis (110 cases/440 controls). The highest point estimate in terms of both pAUC and SN at 98% SP was CA125^EIA^ (pAUC 0.71 (0.66, 0.76), SN at 98% SP 0.29 (0.17–0.42)). When evaluating biomarker combinations, adding CA15-3^STn^ to CA125^EIA^ resulted in an improvement in the point estimate, yielding the highest pAUC (CA125^EIA^ + CA15-3^STn^: 0.75 [0.70–0.80]) and the highest SN at 98% SP (0.32 [0.20–0.47]), although with overlapping confidence intervals. A similar improvement was seen when adding CA15-3^STn^ to the combination of CA125^EIA^ + HE4^EIA^. There seemed to be a synergistic effect between HE4 and the glycovariants, with improved capacity over any single biomarker. However, adding HE4^EIA^ to CA125^EIA^ did not provide a similar synergistic effect

Marker	AUC	95% CI AUC	pAUC	95% CI pAUC	SN at 98% SP	95% CI SN
CA125^MGL^	0.79	[0.73, 0.84]	0.65	[0.6, 0.71]	0.21	[0.13, 0.33]
CA125^STn^	0.75	[0.69, 0.8]	0.65	[0.6, 0.7]	0.17	[0.1, 0.28]
CA15-3^STn^	0.67	[0.62, 0.74]	0.64	[0.6, 0.69]	0.19	[0.1, 0.28]
CA125^EIA^	0.78	[0.72, 0.84]	0.71	[0.66, 0.76]	0.29	[0.17, 0.42]
HE4^EIA^	0.73	[0.67, 0.79]	0.62	[0.57, 0.67]	0.18	[0.1, 0.27]
CA15-3^EIA^	0.63	[0.56, 0.69]	0.57	[0.53, 0.61]	0.10	[0.03, 0.16]

*Abbreviations*:* AUC* Area under the curve, *CI* Confidence interval, *EIA* Enzyme immunoassay, *MGL* Macrophage galactose-type lectin, *pAUC* Partial area under the curve, *SN* Sensitivity, *SP* Specificity, *STn* Sialyl-Thomsen-nouveau

**Fig. 2:**
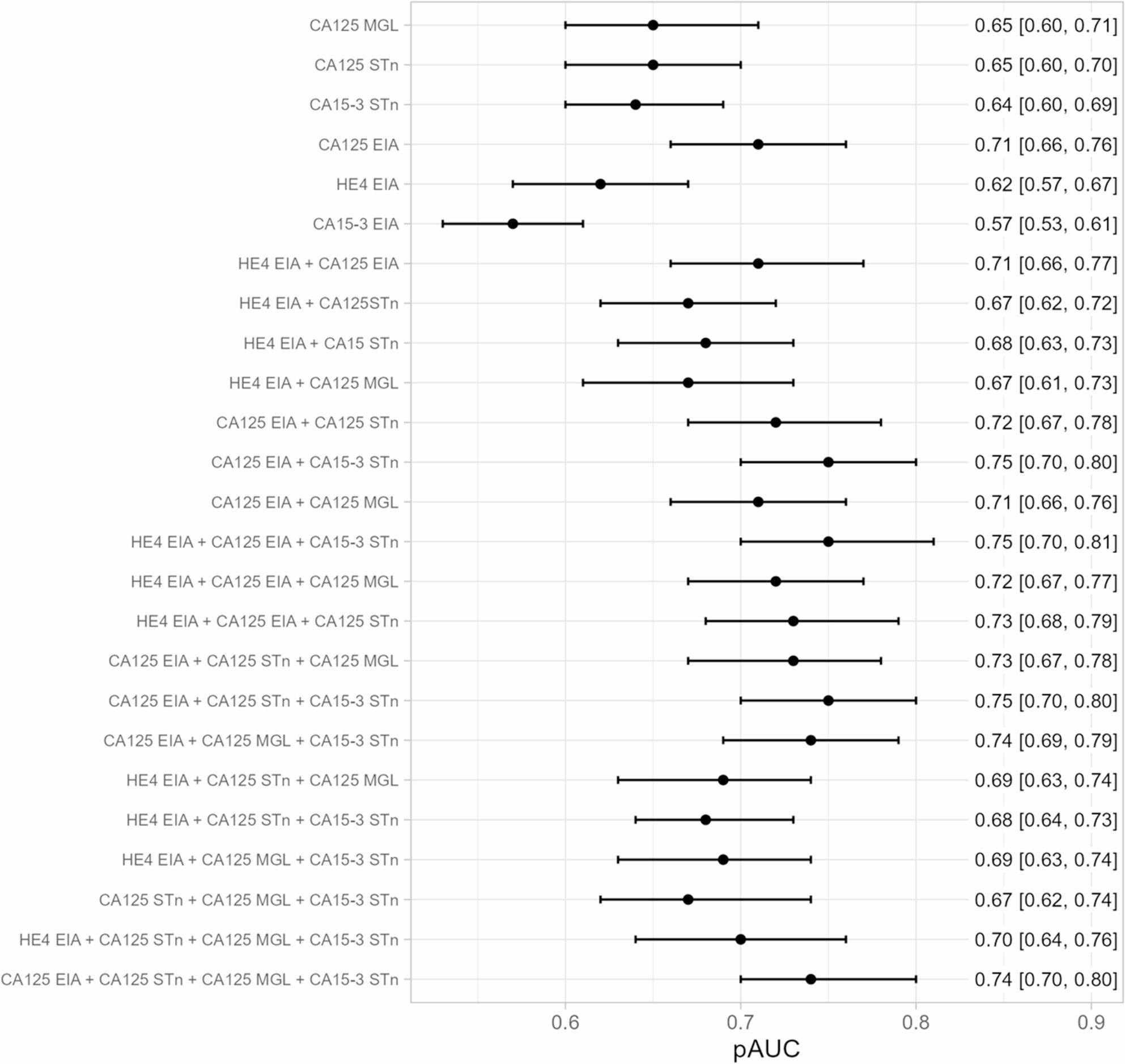
Forest plot presenting pAUC of individual and combined biomarkers in ovarian cancer detection Single and combination biomarker forest plot for pAUC of CA125^MGL^, CA125^STn^, CA15-3^STn^, CA125^EIA^, HE4^EIA^, and CA15-3^EIA^ in samples collected prospectively up to three years prior to epithelial ovarian cancer (*n* = 83) or borderline ovarian tumor (*n* = 27) diagnosis (110 cases/440 controls)

Adding several biomarkers to the combination did not improve performance further (Fig. 2).

**Fig. 3:**
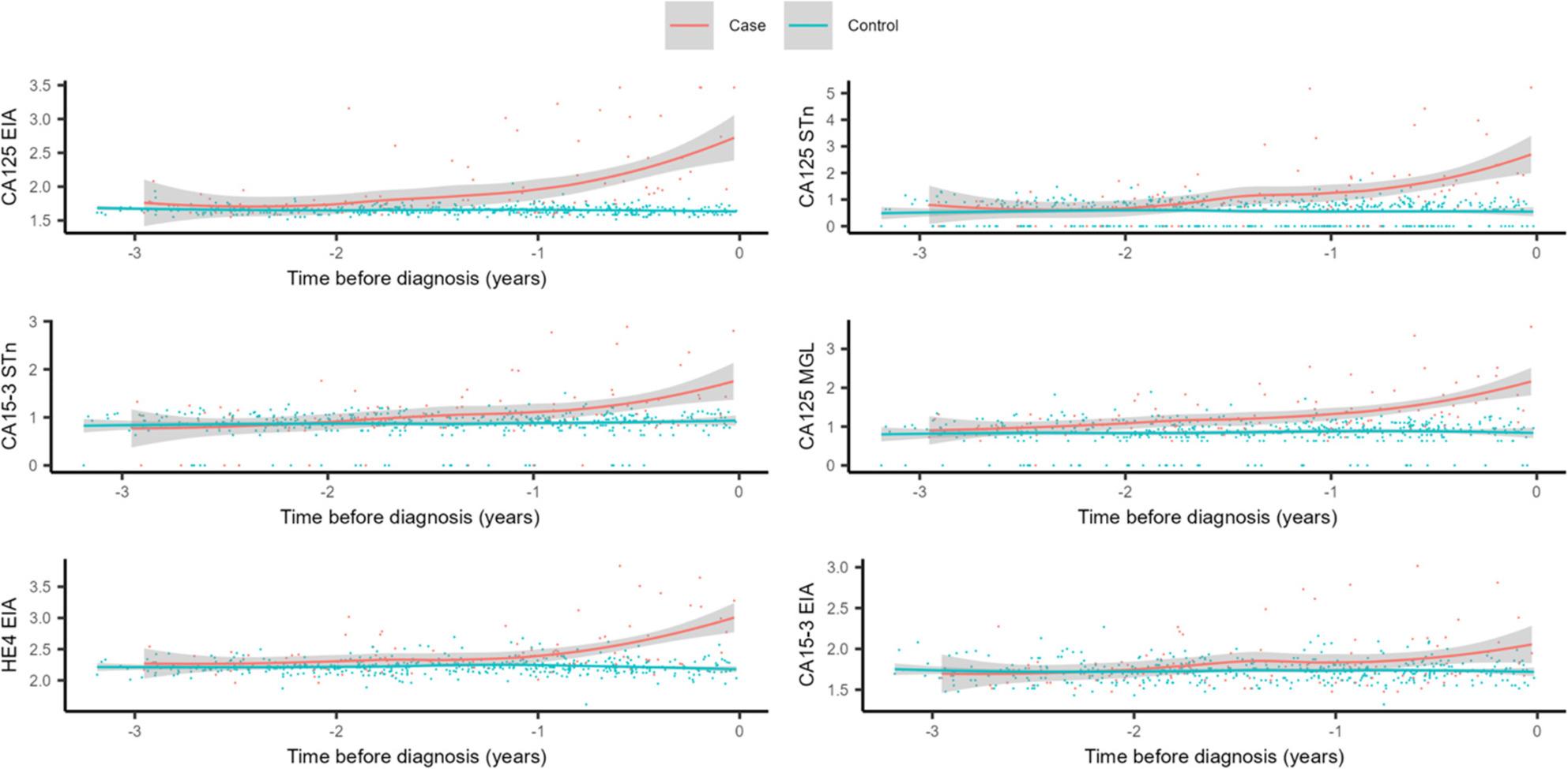
Relative concentrations of glycovariants and standard assays three years prior to ovarian cancer diagnosis Relative concentrations of CA125^MGL^, CA125^STn^, CA15-3^STn^, CA125^EIA^, HE4^EIA^, and CA15-3^EIA^ in 110 cases (epithelial ovarian cancer (*n* = 83), borderline ovarian tumor (*n* = 27)) and 440 matched controls

Loess regression curves were used to visualize the average levels of individual biomarkers 0–3 years prior to EOC or BOT diagnosis, divided by cases and controls. Visually, CA125^EIA^ was the biomarker that rose earliest prior to diagnosis compared to the other reference biomarkers and the glycovariant biomarkers. The earliest rising glycovariant biomarker was CA125^MGL^ (Fig. 3).

The performance of each biomarker across the cohort stratified by lag time is shown in Table 3. For both 0–<1 year and 1–<2 years lag time, the best-performing biomarker that exhibited the highest point estimate for both pAUC and SN at 98% SP was CA125^EIA^ (pAUC 0.84 (0.76, 0.91), SN at 98% SP 0.61 (0.33, 0.77)). For 2–<3 years lag time, CA125^EIA^ had the highest pAUC but HE4^EIA^ had the highest SN at 98% SP.

**Table 3:** Lag-time stratified nanoparticle aided biomarker detection of ovarian cancer Nanoparticle-aided detection of CA125^MGL^, CA125^STn^, CA15-3^STn^, and EIA markers in samples collected prospectively up to three years prior to epithelial ovarian cancer or borderline ovarian tumor diagnosis, stratified by lag time: 0–<1 year (40 cases/160 controls), 1–<2 years (40 cases/160 controls), 2–<3 years (30 cases/120 controls)

Marker	AUC	95% CI AUC	pAUC	95% CI pAUC	SN at 98% SP	95% CI SN
Lag time 0–<1 year						
CA125^MGL^	0.90	[0.83, 0.96]	0.82	[0.72, 0.88]	0.48	[0.33, 0.72]
CA125^STn^	0.88	[0.81, 0.95]	0.82	[0.72, 0.89]	0.51	[0.25, 0.68]
CA15-3^STn^	0.76	[0.64, 0.83]	0.70	[0.63, 0.79]	0.29	[0.15, 0.49]
CA125^EIA^	0.89	[0.81, 0.95]	0.84	[0.76, 0.91]	0.61	[0.33, 0.77]
HE4^EIA^	0.85	[0.76, 0.91]	0.71	[0.64, 0.8]	0.38	[0.15, 0.51]
CA15-3^EIA^	0.73	[0.61, 0.83]	0.64	[0.57, 0.73]	0.17	[0.06, 0.36]
Lag time 1–<2 years						
CA125^MGL^	0.75	[0.67, 0.83]	0.63	[0.56, 0.73]	0.19	[0.08, 0.33]
CA125^STn^	0.68	[0.56, 0.77]	0.61	[0.55, 0.69]	0.15	[0.06, 0.28]
CA15-3^STn^	0.64	[0.5, 0.74]	0.64	[0.5, 0.72]	0.17	[0.05, 0.3]
CA125^EIA^	0.74	[0.62, 0.83]	0.68	[0.61, 0.77]	0.26	[0.08, 0.4]
HE4^EIA^	0.71	[0.6, 0.8]	0.58	[0.53, 0.66]	0.15	[0.04, 0.27]
CA15-3^EIA^	0.62	[0.42, 0.72]	0.58	[0.49, 0.66]	0.10	[0.01, 0.2]
Lag time 2–<3 years						
CA125^MGL^	0.63	[0.53, 0.71]	0.50	[0.48, 0.57]	0.01	[0, 0.07]
CA125^STn^	0.58	[0.47, 0.67]	0.51	[0.47, 0.56]	0.02	[0, 0.05]
CA15-3^STn^	0.42	[0.44, 0.69]	0.49	[0.49, 0.63]	0.01	[0.01, 0.21]
CA125^EIA^	0.64	[0.44, 0.73]	0.57	[0.49, 0.66]	0.06	[0.01, 0.19]
HE4^EIA^	0.60	[0.45, 0.67]	0.55	[0.49, 0.62]	0.07	[0.02, 0.18]
CA15-3^EIA^	0.58	[0.46, 0.64]	0.49	[0.47, 0.54]	0.02	[0, 0.05]

*Abbreviations*: *AUC* Area under the curve, *CI* Confidence interval, *EIA* Enzyme immunoassay, *MGL* Macrophage galactose-type lectin, *pAUC* Partial area under the curve, *SN* Sensitivity, *SP* Specificity, *STn* Sialyl-Thomsen-nouveau

**Fig. 4:**
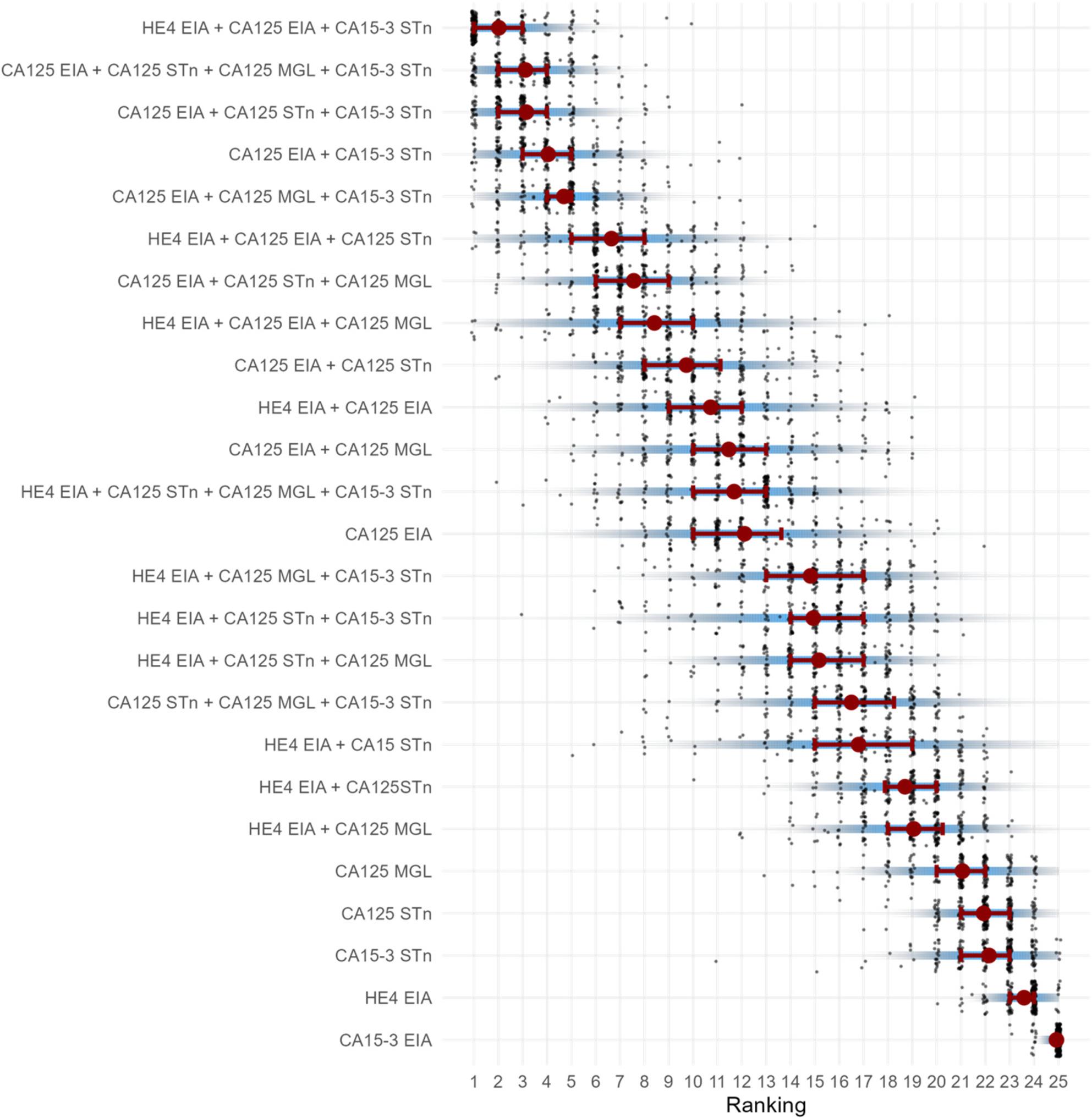
Ranking of individual and combined biomarkers in ovarian cancer detection Ranking figure visualizing the relative performance of biomarkers in samples collected prospectively up to three years prior to EOC or BOT diagnosis (110 cases/440 controls) The performance of the biomarkers with regard to each subgroup – EOC, BOT, HGSC, and ≥ 51 years of age – is shown in the supplementary material. The highest point estimate for single biomarkers (in terms of both pAUC and SN at 98% SP) for EOC was CA125^EIA^, while the best combination was CA125^EIA^ + HE4^EIA^ + CA15-3^STn^ (Figure S1; Table S1). The best point estimate (pAUC, SN at 98% SP) single marker in the BOT subgroup was CA125^EIA^; in the HGSC subgroup, CA15-3^STn^; and in the subgroup ≥ 51 years of age, CA125^MGL^ (Tables S2–4).

Figure 4 visualizes all tested individual and combinations of biomarkers in terms of relative performance of pAUC for detecting EOC and BOT prospectively up to three years before diagnosis. The five best combinations are likely to improve pAUC compared to CA125^EIA^. It is noteworthy that the combination CA125^EIA^ + CA15-3^STn^ was part of all five of the best combinations.

## Discussion

This retrospective population-based case-control study evaluated the discriminatory performance of nanoparticle-aided detection of glycovariants of CA125 and CA15-3 as potential early diagnostic biomarkers of EOC and BOT up to three years prior to diagnosis.

The results presented in this article did not provide evidence of a diagnostic improvement with the glycovariant biomarkers compared with the clinically established biomarker CA125^EIA^. None of the individual glycovariant biomarkers investigated have sufficient potential to alone extend the lead time longer than CA125^EIA^, in the whole cohort or for any of the subgroups investigated.

Previous work indicates that some biomarkers can be detected up to three years prior to EOC diagnosis [24]. More recent data shows that the preclinical detectable phase of protein biomarkers is closer to 12 months which is also supported by the results of this study [25]. In the UK Collaborative Trial of Ovarian Cancer Screening (UKCTOCS) study, multimodal screening was evaluated using repeat CA125^EIA^ testing and a longitudinal CA125^EIA^ algorithm, with transvaginal ultrasound scanning as a second-line test. A stage-shift to lower clinical stage at diagnosis was found, but this did not translate into mortality reduction [26, 27]. In comparison to our study, the UKCTOCS-cohort was significantly larger, with the capacity to investigate stage-shift and mortality benefits with a prospective design. Even with a larger cohort and a well-designed study, use of CA125 did not reduce mortality in combination with transvaginal ultrasound evidencing the need for better screening tools. Several attempts have been made to identify a biomarker that improves on the screening performance of CA125^EIA^, but none have yet been proven to be more effective [6, 9].

A recent study by our group suggested that glycovariants of biomarkers can improve diagnostic performance in both serum and cyst fluid at diagnosis of EOC as compared to CA125^EIA^ and HE4^EIA^ [18, 19] (Jain et al. 2025 manuscript). However, this prospective study showed no significant improvement with glycovariant biomarkers. This may have been partly due to the different control populations: in the previous study, which used samples collected at diagnosis, the controls were symptomatic individuals who were undergoing surgery for benign ovarian conditions. In comparison, the controls in this prospective cohort study were women from the general population who were not diagnosed with cancer during the follow-up period. These differences may explain why the glycovariants performed better than CA125^EIA^ in the diagnostic cohort but not in the prospective cohort; the ability of the glycovariants to discriminate malignant lesions from benign may have been better than that of CA125^EIA^, while CA125^EIA^ has good capacity discriminating malignant lesions from healthy controls [28]. In addition, the controls were younger than the cases in the previous study, but the two groups were of the same age in this prospective study. Although the analyses were age-adjusted in both studies, we cannot rule out the possibility that this affected the results. Notably, in this study the proportion of women with benign ovarian conditions in the control population was likely low, but represents what is to be expected in a true average-risk screening cohort.

In our previous study, the sample matrix was serum, for which the method is optimized.

Serum does not contain anticoagulants, and therefore no anticoagulant-related interferences occur. When using plasma, however, fibrinogen and clotting factors may introduce additional assay interferences. These issues have been successfully addressed through extensive optimization, and the optimized assay buffer was used in the present study. Using this optimized buffer for both serum and plasma, we did not observe any noticeable differences between the matrices, although we cannot completely rule out the possibility that this may have influenced the results.

Studies based on methods that use a panel of biomarkers have been reported to have better sensitivity than single biomarkers with regard to diagnosing early-stage EOC [29]. The combination of HE4 and CA125 has been proven to be more efficient than using the biomarkers individually [30], and is used in a clinical setting in many countries. Adding CA15-3^STn^ to CA125^EIA^ and CA125^EIA^ + HE4^EIA^ further improved the discriminatory capacity in a previous study [18], and a point estimate improvement, although not statistically significant, was also observed in this study. This may be attributable to the improved detection of mucinous cancers with CA15-3^STn^, as previously demonstrated [18], leading to an overall increase in EOC detection rates. Contrary to the results achieved with benign controls in the previous study, HE4^EIA^ did not improve performance over CA125^EIA^ when using “healthy” controls in this cohort.

The diagnostic performance of EIA biomarkers decreases with increasing time between blood draw and cancer diagnosis, which is in line with previous studies [8, 24, 31] and expected for markers associated with tumor development. This is also true for the glycovariant biomarkers used in this study. The small cohort limited the power to detect any differences in biomarker performance. Furthermore, previous studies on the nanoparticle aided assay used serum samples, while in this study we used EDTA plasma, which is the most common matrix in prospective cohorts. Volumes were optimized for the clinical cohort, but for a prospective cohort larger sample volumes are likely to improve detection of very small quantities of biomarkers.

The strengths of this study include the population-based cohort representative of the population of the Västerbotten county, prospective blood samples, and the high quality of the samples used. Laboratory personnel were blinded to case-control status, decreasing the risk of bias. Histopathological diagnoses were validated by a histopathological report review and reassessment of tumor slides, carried out by experienced senior consultant gynecological pathologists. One limitation of the study is that the median age of women with EOC diagnoses was 60.4 years, which is lower than the mean age of 65 years for EOC diagnoses in the Swedish population. This changed the proportion of the different histotypes, e.g. lower proportion of HGSC than expected, and may have caused variation in the results. One reason for the skewed median age in our study is that the blood samples in the VIP study were drawn at the ages 40, 50, and 60, all of which are below the mean age for ovarian cancer. Another limitation of this study is the small sample size, which limited the analysis of the different subgroups, including bootstrap validation of the EOC subgroup, why these results should be interpreted with caution.

## Conclusion

This nested case-control study, which used plasma samples collected up to three years before EOC or BOT, did not provide evidence of a higher discriminatory capacity of the glycovariant biomarkers compared with the clinically established biomarker CA125^EIA^. None of the glycovariant biomarkers investigated had sufficient potential to extend the lead time longer than CA125^EIA^. To efficiently evaluate the performance of the nanoparticle-aided glycovariant detection of EOC and BOT in prospective cohorts, sample volumes greater than the 3 µl used in this study and larger cohorts are needed.

## Supplementary Information

Supplementary Material 1.

## Data Availability

The data used in this study were obtained from the Northern Sweden Health and Disease Study cohort and the County Council of Region Västerbotten after approval by the Swedish Ethical Review Authority. Under Swedish law, individual-level health data containing potentially identifiable and sensitive patient information cannot be shared publicly. Researchers may request access to the data from the corresponding author; such requests will be subject to a formal secrecy assessment in accordance with the Swedish Act on Public Access to Information and Secrecy (2009:400) and applicable ethical approvals.

## Abbreviations

AUC: Area under the receiver operating characteristic curve
BMI: Body mass index
BOT: Borderline ovarian tumor
CA125: Cancer antigen 125
CA15-3: Cancer antigen 15 − 3
CI: Confidence interval
CV%: Coefficient of variation
EIA: Enzyme immunoassay
EOC: Epithelial ovarian cancer
FIGO: International Federation of Gynecology and Obstetrics
HE4: Human epididymis protein 4
HGSC: High-grade serous carcinoma
LGSC: Low-grade serous carcinoma
LOD: Limit of detection
Loess: Locally estimated scatterplot smoothing
mAb: Monoclonal antibody
MGL: Macrophage galactose-type lectin
MONICA: Monitoring Trends and Determinants in Cardiovascular Disease
NSHDS: Northern Sweden Health and Disease Study
pAUC: Partial area under the receiver operating characteristic curve
RT: Room temperature
SA: Streptavidin
SN: Sensitivity
SP: Specificity
STn: Sialyl-Thomsen-nouveau
TRF: Time-resolved fluorescence
VIP: Västerbotten Intervention Programme
WHO: World Health Organization
