# Supplementary material for: Predictive ability of novel glycovariant biomarkers of CA125 and CA15-3 up to three years prior to ovarian cancer diagnosis: a population-based case-control study
Source: J Ovarian Res. 2026 May 29;19:198. doi: 10.1186/s13048-026-02143-5 (PMC13220484; doi:10.1186/s13048-026-02143-5)
Supplement: Supplementary file 1 — Supplementary Material 1. [file 13048_2026_2143_MOESM1_ESM.docx]

**Supplementary data**

**Figure S1.** Forest plot presenting pAUC of individual and combined biomarkers in epithelial ovarian cancer detection

**
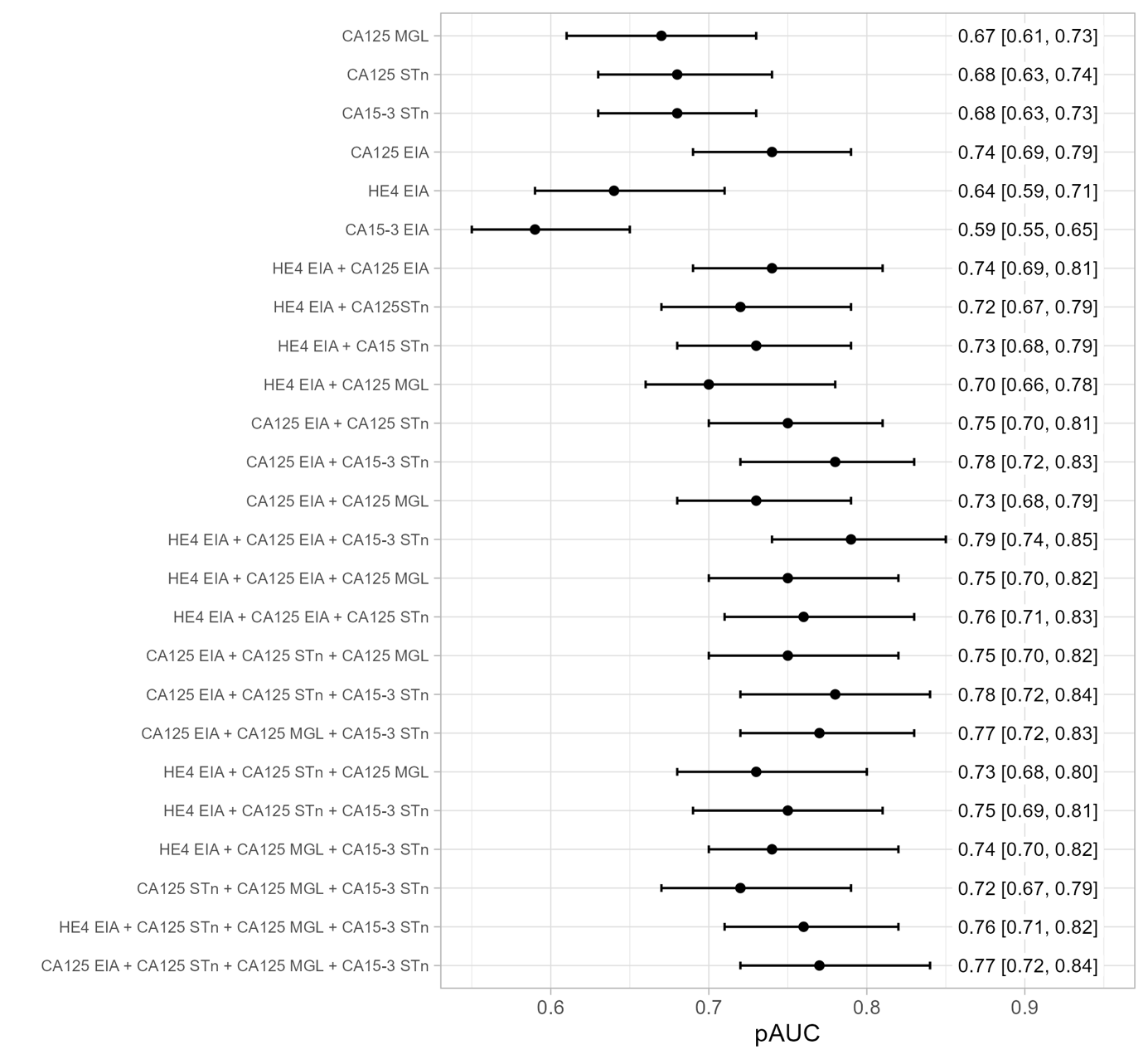
**

Abbreviations: EIA, enzyme immunoassay; MGL, macrophage galactose-type lectin; pAUC, partial area under the curve; STn, Sialyl-Thomsen-nouveau.

Single and combination biomarker forest plot for pAUC of CA125^MGL^, CA125^STn^, CA15-3^STn^,_,_CA125^EIA^, HE4^EIA^, and CA15-3^EIA^ in samples collected prospectively up to three years prior to epithelial ovarian cancer diagnosis (83 cases/332 controls).

**Table S1**. Nanoparticle-aided detection of CA125 and CA15-3 glycovariants, and EIA biomarkers in epithelial ovarian cancer.

| Marker | AUC | 95% CI AUC | pAUC | 95% CI pAUC | SN at 98% SP | 95% CI SN |
| --- | --- | --- | --- | --- | --- | --- |
| CA125^MGL^ | 0.81 | [0.77, 0.87] | 0.67 | [0.61, 0.73] | 0.24 | [0.14, 0.36] |
| CA125^STn^ | 0.78 | [0.72, 0.85] | 0.68 | [0.63, 0.74] | 0.22 | [0.12, 0.36] |
| CA15-3^STn^ | 0.71 | [0.66, 0.79] | 0.68 | [0.63, 0.73] | 0.18 | [0.10, 0.31] |
| CA125^EIA^ | 0.80 | [0.74, 0.87] | 0.74 | [0.69, 0.79] | 0.33 | [0.21, 0.47] |
| HE4^EIA^ | 0.79 | [0.73, 0.85] | 0.64 | [0.59, 0.71] | 0.21 | [0.11, 0.32] |
| CA15-3^EIA^ | 0.66 | [0.59, 0.73] | 0.59 | [0.55, 0.65] | 0.12 | [0.06, 0.21] |

Abbreviations: AUC, area under the curve; CI, confidence interval; EIA, enzyme immunoassay; MGL, macrophage galactose-type lectin; pAUC, partial area under the curve; SN, sensitivity; SP, specificity; STn, Sialyl-Thomsen-nouveau. Samples were collected prospectively up to three years prior to epithelial ovarian cancer diagnosis (83 cases/332 controls).

**Table S2.** Nanoparticle-aided detection of CA125 and CA15-3 glycovariants, and EIA biomarkers in borderline ovarian tumors.

| Marker | AUC | 95% CI AUC | pAUC | 95% CI pAUC | SN at 98% SP | 95% CI SN |
| --- | --- | --- | --- | --- | --- | --- |
| CA125^MGL^ | 0.70 | [0.55, 0.82] | 0.60 | [0.49, 0.72] | 0.14 | [0.01, 0.30] |
| CA125^STn^ | 0.62 | [0.50, 0.75] | 0.56 | [0.49, 0.64] | 0.08 | [0.01, 0.18] |
| CA15-3^STn^ | 0.58 | [0.43, 0.75] | 0.51 | [0.47, 0.63] | 0.03 | [0.00, 0.16] |
| CA125^EIA^ | 0.73 | [0.60, 0.85] | 0.63 | [0.55, 0.74] | 0.13 | [0.02, 0.31] |
| HE4^EIA^ | 0.64 | [0.47, 0.78] | 0.56 | [0.48, 0.69] | 0.09 | [0.01, 0.26] |
| CA15-3^EIA^ | 0.54 | [0.44, 0.71] | 0.55 | [0.47, 0.62] | 0.08 | [0.01, 0.17] |

Abbreviations: AUC, area under the curve; CI, confidence interval; EIA, enzyme immunoassay; MGL, macrophage galactose-type lectin; pAUC, partial area under the curve; SN, sensitivity; SP, specificity; STn, Sialyl-Thomsen-nouveau. Samples were collected prospectively up to three years prior to borderline ovarian tumor diagnosis (27 cases/108 controls).

**Table S3.** Nanoparticle-aided detection of CA125 and CA15-3 glycovariants, and EIA biomarkers in high grade serous carcinoma.

| Marker | AUC | 95% CI AUC | pAUC | 95% CI pAUC | SN at 98% SP | 95% CI SN |
| --- | --- | --- | --- | --- | --- | --- |
| CA125^MGL^ | 0.85 | [0.78, 0.91] | 0.67 | [0.59, 0.77] | 0.24 | [0.07, 0.42] |
| CA125^STn^ | 0.85 | [0.77, 0.92] | 0.69 | [0.62, 0.79] | 0.25 | [0.11, 0.46] |
| CA15-3^STn^ | 0.77 | [0.68, 0.88] | 0.73 | [0.65, 0.82] | 0.29 | [0.10, 0.50] |
| CA125^EIA^ | 0.79 | [0.69, 0.89] | 0.72 | [0.63, 0.81] | 0.27 | [0.11, 0.50] |
| HE4^EIA^ | 0.82 | [0.73, 0.90] | 0.63 | [0.56, 0.74] | 0.20 | [0.06, 0.37] |
| CA15-3^EIA^ | 0.74 | [0.64, 0.84] | 0.64 | [0.57, 0.72] | 0.16 | [0.07, 0.35] |

Abbreviations: AUC, area under the curve; CI, confidence interval; EIA, enzyme immunoassay; MGL, macrophage galactose-type lectin; pAUC, partial area under the curve; SN, sensitivity; SP, specificity; STn, Sialyl-Thomsen-nouveau. Samples were collected prospectively up to three years prior to epithelial ovarian cancer diagnosis, high-grade serous carcinoma (HGSC) subtype (39 cases/156 controls).

**Table S4.** Nanoparticle-aided detection of CA125 and CA15-3 glycovariants, and EIA markers in women ≥51 years.

| Marker | AUC | 95% CI AUC | pAUC | 95% CI pAUC | SN at 98% SP | 95% CI SN |
| --- | --- | --- | --- | --- | --- | --- |
| CA125^MGL^ | 0.81 | [0.76, 0.88] | 0.81 | [0.61, 0.74] | 0.26 | [0.15, 0.39] |
| CA125^STn^ | 0.75 | [0.72, 0.85] | 0.75 | [0.63, 0.74] | 0.22 | [0.15, 0.41] |
| CA15-3^STn^ | 0.69 | [0.62, 0.78] | 0.69 | [0.61, 0.73] | 0.17 | [0.09, 0.32] |
| CA125^EIA^ | 0.78 | [0.73, 0.87] | 0.78 | [0.66, 0.87] | 0.27 | [0.15, 0.45] |
| HE4^EIA^ | 0.75 | [0.70, 0.83] | 0.75 | [0.59, 0.68] | 0.19 | [0.10, 0.33] |
| CA15-3^EIA^ | 0.66 | [0.61, 0.77] | 0.66 | [0.57, 0.68] | 0.15 | [0.07, 0.28] |

Abbreviations: AUC, area under the curve; CI, confidence interval; EIA, enzyme immunoassay; MGL, macrophage galactose-type lectin; pAUC, partial area under the curve; SN, sensitivity; SP, specificity; STn, Sialyl-Thomsen-nouveau. Samples were collected prospectively up to three years prior to ovarian cancer diagnosis in women ≥51 years of age (103 cases/412 controls).
